# Harnessing big data for precision medicine: radiomics based application of nanomaterials in MRI enhancement and multimodal therapy of hepatocellular carcinoma

**DOI:** 10.3389/fimmu.2025.1659180

**Published:** 2025-10-20

**Authors:** Hongbin Shen, Deshui Ran, Jida Zhu, Aoxiao Tao, Kai Zheng, Qing Zhu, Haijun Wang

**Affiliations:** ^1^ College of Traditional Chinese Medicine, Shandong University of Traditional Chinese Medicine, Jinan, China; ^2^ Department of Imaging, Jinan Second People’s Hospital, Jinan, China; ^3^ Second College of Clinical Medicine, Nanchang University, Nanchang, China; ^4^ Department of Imaging Center, Jinan Nanshan People’s Hospital, Jinan, China

**Keywords:** hepatocellular carcinoma, nanomaterials, MRI enhancement, multimodal therapy, AI driven radiomics

## Abstract

Hepatocellular carcinoma (HCC) ranks among the most lethal malignancies worldwide, characterized by its high metastatic potential and poor prognosis. Early and precise detection and diagnosis of HCC remain a major clinical challenge. Magnetic resonance imaging (MRI), as the most widely used noninvasive technique for diagnosing liver diseases, currently suffers from limitations in traditional contrast agents, including low specificity and limited sensitivity, particularly when detecting small lesions. The emergence of nanotechnology offers novel approaches to enhance the diagnostic accuracy and therapeutic efficacy for HCC. Under the framework of big data driven precision medicine, this study explores the application of nanomaterials in HCC MRI enhancement and multimodal therapy. This review comprehensively summarizes two types of responsive nanomaterials: (1) Chiral Ni(OH)_2_ nanoparticles, which suggeste enhanced contrast in T1 weighted MRI and selective imaging capabilities for primary HCC and lung metastases; (2) β Lapachone loaded mesoporous MnO_2_ nanoparticles (HLMn), which effectively enhance the generation of reactive oxygen species (ROS) within tumor cells, disrupt redox homeostasis, and significantly improve the efficacy of chemo dynamic therapy (CDT). These nanoplatforms also exhibit potential to activate the c-GAS STING innate immune pathway, thereby augmenting antitumor immune responses. Nanomaterials hold great promise not only as enhanced contrast agents but also as precise therapeutic carriers. By integrating radiomics based imaging features with biological markers, we summarize current personalized HCC diagnosis and treatment planning models based on multimodal data. Simultaneously, we provide a critical summary of the synergistic application of advanced imaging and therapeutic nanotechnologies. In the future, leveraging big data for precise HCC diagnosis and treatment is anticipated to significantly improve patient survival.

## 1 Introduction and literature search strategy

### 1.1 Introduction

Hepatocellular carcinoma (HCC) is one of the most common and deadly malignant tumors globally, accounting for the majority of primary liver cancers (1). Due to its asymptomatic nature in early stages, rapid progression, and high metastatic potential, early and accurate diagnosis of HCC remains challenging (2). Consequently, patients often present at advanced stages, resulting in poor prognosis and limited treatment options (3, 4). Current diagnostic modalities, including ultrasound, computed tomography (CT), and magnetic resonance imaging (MRI), along with serum biomarkers such as alpha fetoprotein (AFP) and des γ carboxy prothrombin (DCP), face challenges such as limited sensitivity for small or early stage tumors, inability to fully capture tumor heterogeneity, and variability in detecting microvascular invasion. Similarly, conventional therapeutic approaches, including surgical resection, local ablation, transarterial chemoembolization (TACE), and systemic chemotherapy, often result in suboptimal treatment responses due to tumor heterogeneity and complex tumor microenvironment, contributing to the overall poor prognosis (5). While significant progress has been made in treatment modalities such as surgical resection, transarterial chemoembolization (TACE), and systemic chemotherapy, the five year survival rate remains suboptimal, with rates in many regions barely exceeding 30% (6). These factors underscore the urgent need to improve early detection and precision treatment to enhance patient outcomes and reduce mortality.

Magnetic resonance imaging (MRI) has become the gold standard for non-invasive liver cancer diagnosis due to its excellent spatial resolution, high soft tissue contrast, and multi parameter detection capabilities, enabling simultaneous detection of anatomical and functional abnormalities in liver tissue (7). However, traditional gadolinium based contrast agents have limitations such as low sensitivity, lack of tumor specificity, and potential nephrotoxicity. These limitations further highlight the need for advanced imaging strategies that can provide higher sensitivity, tumor specific contrast, and reliable quantitative biomarkers. Nanotechnology, as a highly promising research direction, suggests significant potential in biomedical applications due to its customizable size, shape, surface charge, and chemical composition (8). When applied to MRI, it can significantly enhance T1 or T2 contrast, improve tumor targeting, and serve as a delivery system for targeted therapeutic drugs (9, 10). Functionalized nanomaterials can detect overexpressed biomarkers on HCC cell membranes, such as glycoprotein 3 and CD44, achieving selective distribution to tumor sites while minimizing off target effects (11).

The emergence of radiomics (the efficient extraction of quantitative features from medical images) has opened up new dimensions for precision oncology. Research has shown that radiomics can analyze tumor heterogeneity, morphological changes, and the composition of the tumor microenvironment, while also revealing features that are not detectable by the naked eye. When combined with Machine Learning (ML) and Deep Learning (DL) approaches, radiomics enables integration of multi-dimensional imaging data with clinical, pathological, and molecular information, providing the theoretical and computational basis for AI driven predictive modeling of individualized treatment responses. This framework supports the rationale for leveraging nanomaterials to enhance imaging contrast and guide precise, personalized therapies. Additionally, the integration of image guided, data driven nanotechnology with MRI marks a paradigm shift in cancer treatment planning and optimization (12). This review aims to explore the application of nanomaterials in enhancing MRI contrast and promoting multimodal treatment for hepatocellular carcinoma (HCC) (13). In the future, by overcoming the limitations of current diagnostic and therapeutic methods and fully exploiting the capabilities of nanotechnology and radiomics, more accurate and personalized tools for HCC diagnosis and treatment can be established, ultimately improving patient outcomes.

### 1.2 Literature search strategy

To ensure a comprehensive and systematic review of nanomaterials in hepatocellular carcinoma (HCC) imaging and therapy, we conducted a thorough literature search across major scientific databases, including PubMed, Web of Science, and Scopus, covering publications up to May 2025. The search strategy combined terms related to the disease, imaging modalities, nanomaterials, and therapeutic approaches, including: *“hepatocellular carcinoma”*, *“HCC”*, *“magnetic resonance imaging”*, *“MRI”*, *“nanomaterials”*, *“nanoparticles”*, *“radiomics”*, *“multimodal therapy”*, *“photothermal therapy”*, and *“chemodynamic therapy”*. Boolean operators (“AND”, “OR”) and truncation were applied to refine search results and ensure maximum coverage of relevant literature.

Inclusion criteria were: (1) Studies involving HCC patients or preclinical models relevant to human HCC. (2) Research focusing on nanomaterial-based MRI contrast enhancement, targeted drug delivery, or multimodal therapy. (3) Studies providing quantitative, mechanistic, or translational insights. (4) Peer-reviewed original research articles published in English.

Exclusion criteria included: (1) Articles not directly related to HCC or nanomaterials. (2) Conference abstracts, editorials, commentaries, or reviews without primary data. (3) Duplicate studies or studies with insufficient methodological details.

The initial search yielded over 1,200 publications. After screening titles and abstracts for relevance and removing duplicates, 320 articles were selected for full-text review. Each full-text article was evaluated for methodological rigor, experimental design, and clinical or preclinical relevance. Ultimately, 101 studies were included in this review, providing a comprehensive and balanced representation of current research on nanomaterial applications in HCC imaging and therapy.

To further enhance comprehensiveness, the reference lists of included studies were manually screened to capture additional relevant publications not indexed in the databases. Priority was given to recent studies (2020–2025) and highly cited works that provide mechanistic insights or novel applications.

For synthesis and analysis, the included studies were categorized based on key criteria: (1) type of nanomaterial (e.g., manganese-based, iron oxide, gold nanoparticles), (2) imaging modality and contrast enhancement mechanism, (3) therapeutic strategies (e.g., photothermal therapy, chemodynamic therapy, immunomodulation), and (4) preclinical versus clinical studies. Comparative tables and critical discussions were constructed to highlight the strengths, limitations, and translational potential of each approach. This structured methodology ensures that the review provides not only a comprehensive overview but also critical insights and guidance for future research directions in HCC nanomedicine.

## 2 Radiomics basis of MRI enhancement by nanomaterials in HCC imaging

MRI plays a significant role in assisting HCC diagnosis. Recent studies have integrated nanomaterials into hepatocellular carcinoma imaging technology, primarily to extract key features from medical imaging data and efficiently transmit this data to achieve a visual format suitable for further analysis. Additionally, the functionalized nanoparticles introduced during imaging provide critical features such as enhanced contrast, tumor specific distribution, and time and space specific interactions, which are essential for subsequent applications in radiomics (14). Advances in multimodal imaging technology have broken through the limitations of traditional imaging, meeting the current demands of precision medicine, and significantly promoting the detection of phenotypic patterns in the tumor microenvironment. In the future, the integration of nanomaterials with radiomics will further enhance diagnostic and prognostic capabilities, thereby solidifying MRI’s position as an indispensable clinical driver in HCC management (15, 16).

### 2.1 Synthesis of nanomaterials

The synthesis of nanomaterials plays a crucial role in biomedicine, particularly in the field of HCC imaging and treatment. This review explores three primary synthesis methods: vapor phase synthesis, solution phase synthesis, and solid phase synthesis. Vapor phase synthesis includes techniques such as physical vapor deposition (PVD) and chemical vapor deposition (CVD), which have been found to effectively prepare nanomaterials with high structural purity and morphological control (17). This method has been successfully applied to the preparation of magnetic nanomaterials for imaging applications. Liquid phase synthesis has become the most flexible method for biomedical applications due to its ease of operation, scalability, and high functionalization rate (18, 19). Studies have shown that precise control of particle morphology can be achieved by adjusting variables such as pH, temperature, and reaction time. Importantly, surface functionalization the grafting of targeted ligands or therapeutic molecules has been successfully achieved during synthesis, which is crucial for the tumor specific application of HCC (20). For example, MRI contrast agents and drug loaded nanocarriers. Solid phase synthesis, including techniques such as ball milling and spark plasma sintering, can produce crystalline nanoparticles with high structural integrity. Although this method is not suitable for surface modification, it offers significant advantages in preparing robust and scalable materials with high thermal and chemical stability (21).

This figure illustrates the synthesis methods of nanomaterials and their applications in biomedical research, particularly in imaging and treatment of HCC. The figure is divided into two main sections: Section (A) shows different synthesis methods and their corresponding material size ranges, while Section (B) provides detailed descriptions of the specific techniques and control parameters involved in each synthesis method.

In Section A of Figure, two methods for synthesizing nanomaterials are presented: bottom up and top-down approaches. The bottom-up method starts with small molecules or atoms (size < 1 nm) and gradually constructs nanostructured materials (1 nm–100 nm) through controlled growth and nucleation processes. This method is closely related to techniques such as hydrothermal synthesis, sol gel synthesis, electroplating, and chemical vapor deposition (CVD). The top-down method, on the other hand, starts with micron sized or bulk materials (size > 1 μm) and reduces them to nanoscale materials through processes such as ball milling and laser processing.

In Section B of Figure, the three main synthesis methods are detailed. For gas phase synthesis, we list techniques such as physical vapor deposition (PVD) and chemical vapor deposition (CVD), as well as sub methods like sputtering and ultra-high vacuum CVD. Solution phase synthesis includes hydrothermal/solvothermal methods, precipitation/coprecipitation methods, and sol gel or electrodeposition methods. Here, particular emphasis is placed on parameters such as pH, temperature, and concentration control. Solid phase synthesis encompasses both ambient and high temperature processes, including key techniques such as mechanical exfoliation and solid state grinding.

Additionally, the figure highlights the importance of controlling various parameters during synthesis to achieve the desired properties of nanomaterials. In solution phase synthesis, factors such as pH, temperature, concentration, and component ratios are critical for regulating the structure and functionality of nanomaterials. This level of control is crucial for the application of nanomaterials in biomedical applications.

**Figure 1:**
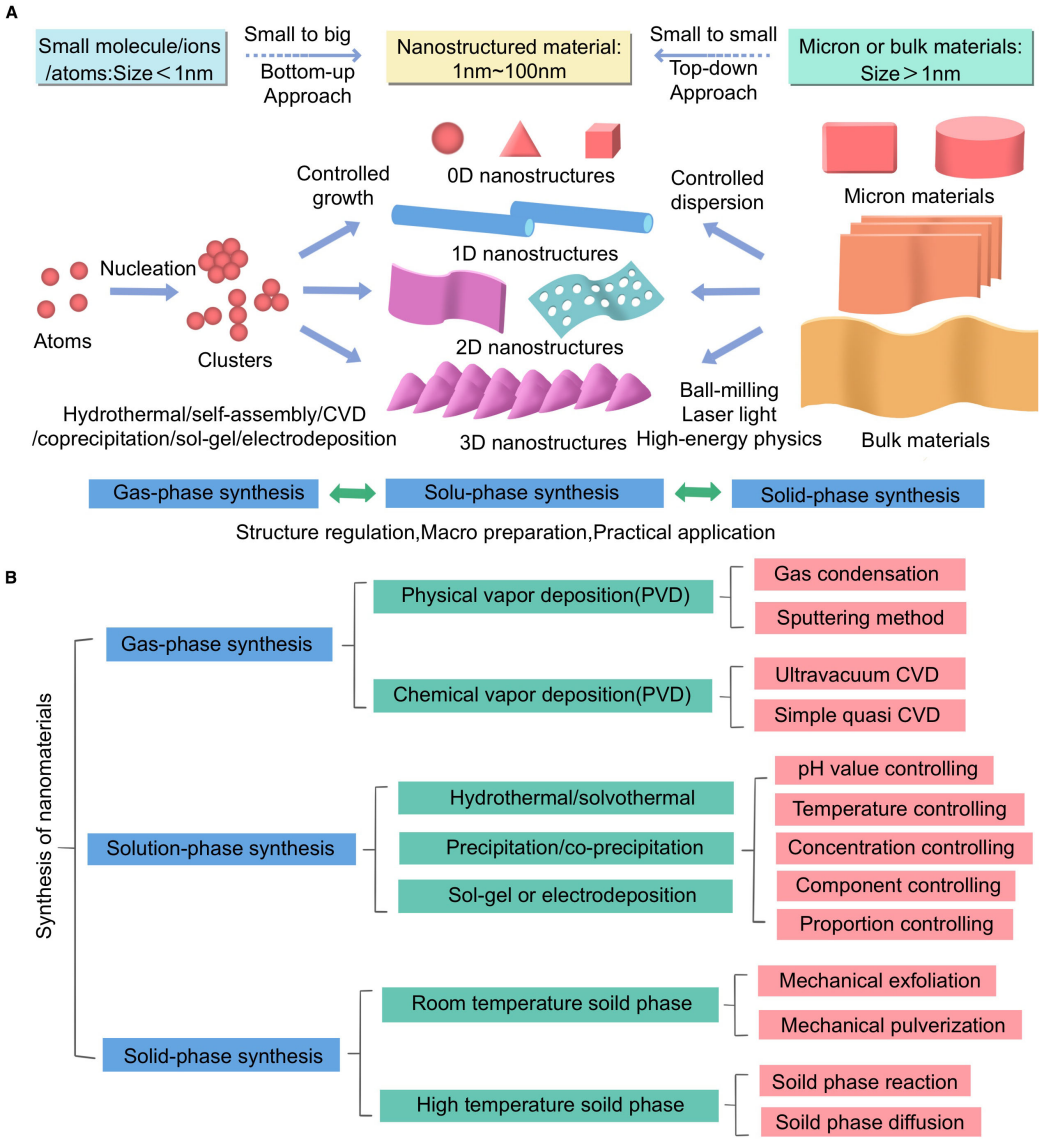
**(A, B)** Synthesis methods of nanomaterials.

This figure aims to comprehensively summarize the complexity and diversity of nanomaterial synthesis methods, emphasizing the importance of selecting appropriate synthesis techniques based on the specific requirements of biomedical applications. It also highlights the potential of these nanomaterials in advancing HCC diagnostic and therapeutic strategies (**Figure 1**).

### 2.2 Mechanisms and advantages of nanomaterials in MRI imaging

Integrating magnetic resonance imaging (MRI) into nanomaterials by adjusting their magnetic and physicochemical properties can significantly improve the diagnostic accuracy of HCC. Although gadolinium-based contrast agents have seen some clinical use, their limited tumor specificity, low sensitivity, and potential nephrotoxicity continue to pose significant barriers to widespread adoption. In contrast, nanomaterials offer significant advantages in terms of magnetic responsiveness, biocompatibility, and surface functionalization, making them promising candidates for next generation MRI contrast agents (22).

Current research focuses on chiral nickel hydroxide nanoparticles [D/L Ni(OH)_2_], which exhibit a high longitudinal relaxation rate (r_1_), directly influencing T1 relaxation time. The enhancement in T1 weighted imaging stems from these nanoparticles increasing proton relaxation by modulating the local magnetic field, thereby amplifying the MRI signal (23). The chiral configuration also introduces stereoselective interactions with cellular components. DNi(OH)_2_ exhibits stronger affinity for specific tumor receptors, resulting in higher cellular uptake and more pronounced signal amplification in tumor regions compared to LNi(OH)_2_ or achiral analogues. This chiral selectivity enhances spatial resolution and tumor specificity, representing a significant advancement over traditional drugs.

Manganese dioxide nanoparticles (MnO_2_) are another class of effective T1 contrast agents (24). In the acidic and reducing tumor microenvironment (TME), MnO_2_ undergoes redox reactions, releasing Mn²^+^ ions. These Mn²^+^ ions exhibit paramagnetic properties, significantly shortening T1 relaxation times through interactions with water protons, thereby enhancing signal intensity in T1 weighted MRI sequences. Furthermore, MnO_2_ particles have an inherent ability to react with endogenous hydrogen peroxide (H_2_O_2_), which is commonly overexpressed in tumor tissues. This catalyzed reaction generates oxygen (O_2_), alleviating tumor hypoxia and subsequently enhancing the efficacy of oxygen dependent treatments such as photodynamic therapy (PDT) (25). Manganese dioxide (MnO_2_) exhibits dual functionality in enhancing imaging contrast and modulating the tumor microenvironment (TME), which aligns closely with the principles of theranostics. Both D Ni(OH)_2_ and MnO_2_ nanoparticles can be further modified with tumor targeting ligands to enhance selectivity and systemic circulation time (26, 27). Ligands such as hyaluronic acid (HA) targeting the CD44 receptor or tumor specific peptides can be grafted onto the nanoparticle surface to achieve active targeting (28). This engineering strategy significantly enhances drug accumulation at the tumor site by enhancing the permeability retention effect and active targeting mechanism. Additionally, the structural parameters of these nanomaterials (including particle size, zeta potential, and hydrophilicity) can be finely tuned to further optimize biodistribution, cellular uptake, and MRI signal intensity. Furthermore, smaller nanoparticles (<50 nm) typically exhibit better tumor tissue penetration, while surface polyethylene glycolation (PEGylation) significantly improves systemic stability and reduces immune clearance (29, 30).

**Figure 2:**
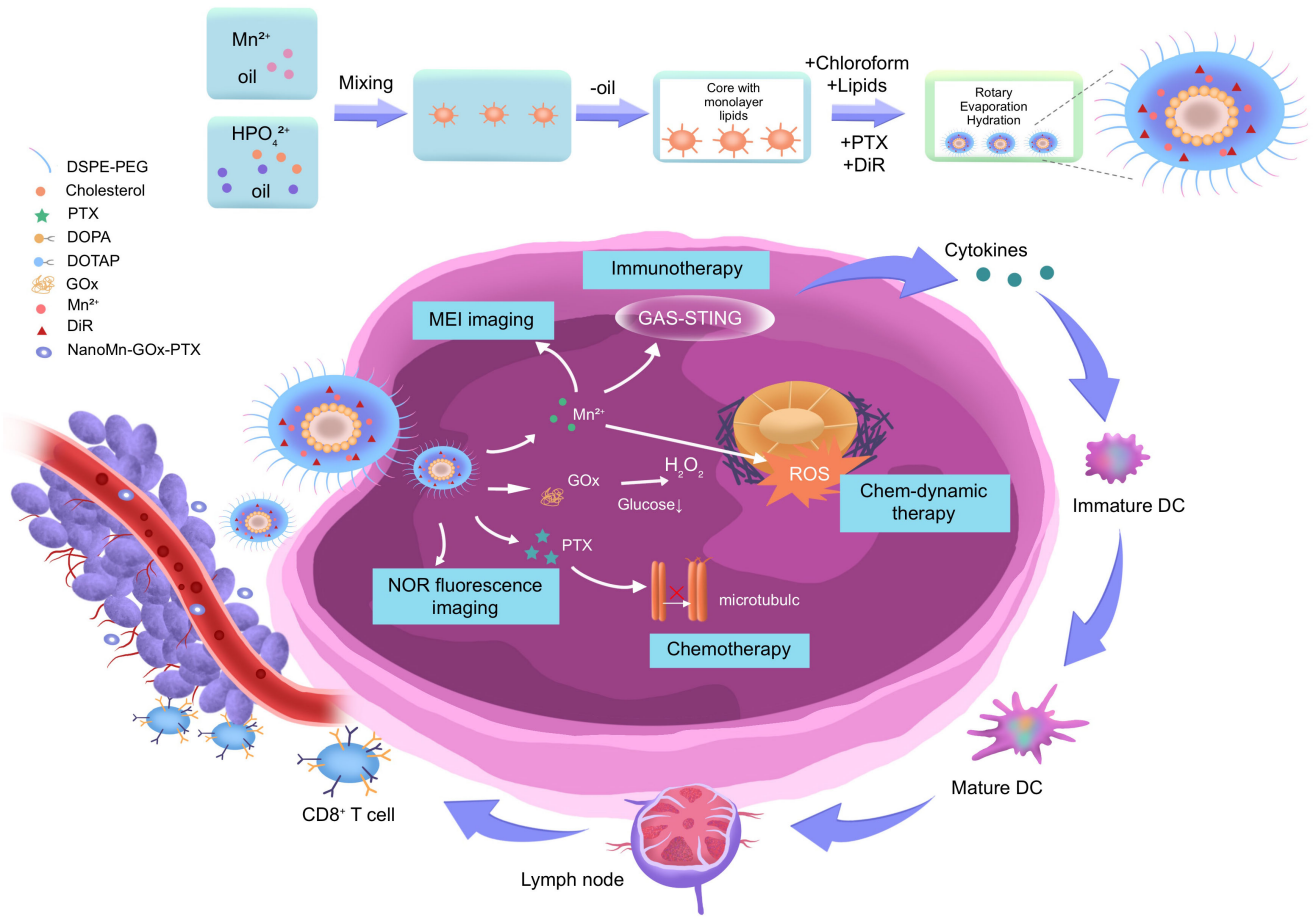
Schematic diagram of the synthesis and anti-tumor of NanoMn Gox drug delivery platform.

To visually illustrate these advantages, we have added representative MRI scans in **Figure 2**, comparing conventional gadolinium-based contrast MRI with nanomaterial enhanced MRI (D Ni(OH)_2_ and MnO_2_). **Figure 2** shows preclinical T1 weighted imaging of HCC xenografts with MnO_2_ nanoparticles, highlighting enhanced tumor contrast and spatial resolution. These figures support the discussion of the unique imaging benefits provided by functionalized nanomaterials.

MRI contrast agents based on nanomaterials (such as D Ni(OH)_2_ and MnO_2_) provide a multifunctional platform with high relaxation rates, environmental responsiveness, and surface tunability. In the future, they could enable high resolution, high contrast, and tumor specific imaging of HCC, thereby further advancing precise diagnosis of HCC.

### 2.3 Feature extraction and diagnostic value of radiomics in HCC MRI

Radiomics, as an emerging discipline, is currently situated at the intersection of medical imaging and data science. In the context of HCC, radiomics offers a non-invasive method for analyzing tumor characteristics such as shape, texture, intensity distribution, and spatial heterogeneity. These features are primarily extracted from MRI sequences such as T1 weighted, T2 weighted, diffusion weighted imaging (DWI), and contrast enhanced imaging, and can help identify key biomarkers associated with tumor biology and clinical outcomes (31).

Radiomic analysis commonly involves the calculation of first order statistics (e.g., mean intensity, entropy), second order texture features (e.g., gray level cooccurrence matrix [GLCM] metrics), and higher order transformations (e.g., wavelet decomposition) from defined regions of interest (ROIs) (32). These data can reflect underlying tumor phenotypes such as vascularity, necrosis, or fibrotic changes, which are relevant in the clinical stratification and staging of HCC. To enhance clinical relevance, we have integrated a detailed workflow outlining the steps of image preprocessing, ROI segmentation, feature extraction, and feature selection, emphasizing how each step can impact downstream predictive modeling and reproducibility (33).

While some studies have suggested associations between radiomic features and prognostic indicators, such as microvascular invasion (MVI), tumor grade, or recurrence risk (34), the reproducibility of these findings remains an active area of investigation. ML methods, including LASSO regression, random forests, support vector machines, and DL approaches, are increasingly employed to identify robust feature subsets and construct predictive models for tumor diagnosis, prognosis, and therapy response prediction. Comparative analyses showing performance differences between conventional MRI features and nanomaterial enhanced MRI features are included to highlight the added diagnostic value of nanomaterial enhanced imaging (35).

Radiomics can also be integrated with conventional biomarkers, such as alpha fetoprotein (AFP), or molecular data (e.g., TP53 mutation status) to enhance diagnostic accuracy and biological interpretation (36). Multi parametric models combining radiomic features with clinical, genomic, and treatment data are now emphasized as a big data driven approach to improve individualized risk stratification and treatment planning (37). For instance, multi parametric models combining radiomic features with clinical data have shown improved performance in distinguishing HCC from benign hepatic lesions like focal nodular hyperplasia (FNH) or hemangiomas (38). However, such approaches are still largely experimental and not yet adopted in routine clinical practice.

A major challenge limiting clinical translation lies in the lack of standardization in image acquisition, segmentation protocols, and feature computation (39). Inter scanner variability and institution dependent imaging parameters can significantly affect feature stability and model generalizability. Consequently, we stress the importance of harmonized radiomics pipelines, large annotated datasets, and integration with AI driven predictive modeling for robust, reproducible clinical applications (37).

In summary, radiomics holds considerable potential to complement MRI in the diagnosis and risk stratification of HCC. Although many of its current applications remain investigational, the integration of radiomics with big data analytics, ML, and nanomaterial enhanced imaging represents a promising strategy to improve non-invasive tumor assessment and guide individualized clinical decisions in precision medicine (40).

### 2.4 Integration strategies for multimodal imaging and clinical data

In the era of precision medicine, the integration of radiomics derived imaging features with clinical, pathological, molecular, and therapeutic response data is essential to build robust, data driven diagnostic and therapeutic decision-making frameworks (41). For hepatocellular carcinoma (HCC), which presents with heterogeneous biological behavior, combining MRI based radiomics with genomics, transcriptomics, proteomics, and biochemical markers provides a multidimensional perspective that enhances individualized disease assessment and patient stratification (42).

Tumor heterogeneity and microstructural changes can be quantitatively captured by extracting radiomic features from MRI sequences (e.g., T1 weighted, T2 weighted, and diffusion weighted imaging), which are then correlated with gene expression profiles, mutation status (e.g., TP53, CTNNB1), histological grading, and serum biomarkers such as alpha fetoprotein (AFP) and des γ carboxy prothrombin (DCP) (43, 44). This multidimensional integration facilitates more accurate prognostic stratification and individualized therapy planning.

By incorporating advanced computational techniques, including ML algorithms, deep neural networks, and multi modal data fusion frameworks, these heterogeneous datasets can be analyzed to identify predictive patterns and optimize treatment strategies. Different methods such as feature level fusion, decision level fusion, and model ensemble approaches can bring multiple data types together without redundant information while maintaining model interpretability (45).

Clinical Decision Support Systems (CDSS) are increasingly leveraging these fused data to provide oncologists with real time, evidence-based recommendations for diagnosis, risk assessment, and treatment selection (46). These systems enable dynamic, patient specific decision making based on tumor characteristics, molecular profiles, and risk factors, thereby supporting precision oncology in clinical practice.

Overall, the combination of imaging, molecular, and clinical data transforms traditional diagnostics into a systemic, data driven framework, enhancing HCC diagnosis, patient stratification, and personalized follow up strategies.

## 3 Mechanistic analysis and strategic design of nanomaterial mediated multimodal synergistic therapy

### 3.1 ROS amplification and mechanism of chemodynamic therapy

A study delved into the mechanistic analysis of nanomaterial mediated multimodal synergistic therapy for hepatocellular carcinoma (HCC), with a focus on ROS amplification and the integration of chemo dynamic therapy (CDT) with other therapeutic modalities (47). The β Lapachone (β Lap)/MnO_2_ nanoplatform was found to significantly enhance CDT through cascade ROS augmentation. When activated in the tumor microenvironment, it produces highly toxic hydroxyl radicals (•OH) via Fenton like reactions, inducing oxidative stress and mitochondrial dysfunction, and ultimately causing cancer cell apoptosis (48, 49). In addition, another research also developed a tandem nanoplatform integrating photothermal therapy (PTT), photodynamic therapy (PDT), and CDT. The NanoMn-Gox-PTX system, encapsulated in a DSPE-PEG lipid layer and containing Mn²^+^, glucose oxidase (GOx), and paclitaxel (PTX), suggested remarkable therapeutic and imaging capabilities (50). Upon reaching the tumor tissue, GOx catalyzes the oxidation of glucose to H_2_O_2_, which then reacts with Mn²^+^ to form •OH, triggering CDT (51). Meanwhile, the nanoplatform is activated by near infrared II (NIR II) laser irradiation, elevating local temperature to induce tumor cell apoptosis through PTT (52). The *in situ* production of oxygen improves PDT efficacy by reducing hypoxia induced resistance.

Furthermore, the release of Mn²^+^ enhances T1 weighted MRI contrast and facilitates real time imaging of nanoparticle distribution and therapeutic response through DiR molecules for NIR fluorescence imaging (53). Increased ROS levels upregulate tumor antigen release, activate the cGAS–STING immune axis, and promote dendritic cell maturation and cytotoxic T lymphocyte induction (54). The NanoMn-Gox-PTX nanoplatform, with its rational design and multifunctional capabilities, stands out as an ideal candidate for effective and precise image guided cancer treatment. The β Lap/MnO_2_ nano system offers a promising approach to achieving selective and efficient HCC treatment based on enhanced CDT and MRI detectability, aligning with the study’s objective of developing advanced nanomaterials to overcome traditional HCC treatment limitations (55).

### 3.2 Design and optimization of photothermal/photodynamic synergistic therapy system

Synergistic photothermal/photodynamic therapy (PTT/PDT) systems represent a novel strategic approach for HCC treatment that leverages the complementary advantages of multiple therapeutic modalities (56). The NanoMn-Gox-PTX platform integrates photothermal therapy (PTT), photodynamic therapy (PDT), and chemotherapy driven therapy (CDT) into a single nanostructure, thereby enabling precise drug delivery, real time imaging, and enhanced therapeutic efficacy.

Another nanoplatform employs a DSPE-PEG lipid bilayer to encapsulate components such as manganese ions (Mn²^+^), glucose oxidase (GOx), paclitaxel (PTX), and the fluorescent dye DiR (57). While ensuring near perfect biocompatibility and circulatory stability, it can achieve controlled, precise release under tumor specific stimulation. Following systemic administration, the nanoparticle platform passively accumulates in tumor tissues via the enhanced permeability and retention (EPR) effect (58). Once localized in the tumor microenvironment (TME), the platform automatically responds to its unique biochemical conditions and external irradiation. Mechanistically, GOx catalyzes the oxidation of intratumoral-glucose to gluconic acid and hydrogen peroxide (H_2_O_2_), leading to two critical outcomes (59): (1) depletion of glucose disrupts tumor cell metabolism and promotes starvation induced apoptosis; (2) the generated H_2_O_2_ reacts with Mn²^+^ via a Fenton like reaction to yield highly cytotoxic hydroxyl radicals (•OH), which induce oxidative stress and cellular damage—constituting the CDT component (60).

Simultaneously, under near infrared II (NIR II) laser irradiation, the nanostructure absorbs light and converts it into heat, elevating local temperatures to trigger apoptosis in cancer cells through PTT (61). This localized hyperthermia not only disrupts cellular integrity but also increases membrane permeability, facilitating drug penetration and potentiating chemotherapy. Furthermore, the elevated temperature accelerates ROS production, synergistically enhancing PDT. In the PDT mechanism, the DiR dyeactivated by NIR irradiation—transfers energy to surrounding oxygen molecules to produce singlet oxygen (^1O_2_), a potent ROS that damages intracellular organelles and DNA (62). However, the hypoxic nature of solid tumors often impairs PDT efficacy. To counter this, MnO_2_ reacts with H_2_O_2_ to generate oxygen *in situ*, thereby alleviating hypoxia and supporting continuous ROS generation (63). In terms of imaging, Mn²^+^ released from the platform improves T1 weighted MRI contrast due to its strong paramagnetic properties, enabling real time localization of the nanoplatform and monitoring of treatment progression (64). Concurrently, DiR fluorescence imaging supports near infrared visualization of nanoparticle biodistribution and tumor response.

The schematic diagram illustrates the modular design and mechanistic interactions of the NanoMn-Gox-PTX nanoplatform for hepatocellular carcinoma (HCC) therapy. The figure outlines each stage of the nanoparticle’s fabrication process, beginning with the integration of therapeutic and diagnostic components—including manganese ions (Mn²^+^), DSPE PEG for enhanced stability and biocompatibility, hydrogenated poly(glycerol) (HPG), paclitaxel (PTX), cholesterol PEG, cationic lipid DOTAP, magnetically active material MAG, and the near infrared fluorescent dye DiR. These components are co assembled via molecular lipid core formation, followed by chloroform evaporation and aqueous phase hydration to yield a stable, uniform nanostructure. This formulation strategy ensures precise control over particle size, zeta potential, and encapsulation efficiency, all critical parameters influencing tumor penetration and systemic circulation time.

Following intravenous administration, the nanomaterials passively accumulate in tumor tissues via the enhanced permeability and retention (EPR) effect. Once successfully localized, the platform is functionally activated through endogenous tumor microenvironment stimulation and external near infrared II (NIR II) laser irradiation. At this point, in the tumor site, glucose oxidase (GOx) catalyzes glucose oxidation, producing hydrogen peroxide (H_2_O_2_) and inducing glucose starvation (65). This metabolic effect makes tumor cells more sensitive to further treatment. The generated H_2_O_2_ acts as a substrate for a Fenton like reaction with Mn²^+^, producing highly reactive hydroxyl radicals (•OH), which trigger chemotherapy driven therapy (CDT) through DNA damage and oxidative stress (66). Simultaneously, the release of Mn²^+^ enhances T1 weighted MRI signal intensity, enabling real time anatomical localization and monitoring of therapeutic progression. DiR encapsulated in the lipid bilayer supports NIR fluorescence imaging, offering dynamic tracking of nanoparticle distribution and therapeutic response.

Laser triggered photothermal conversion elevates the local temperature of the tumor microenvironment, inducing direct thermal ablation of malignant cells and improving tumor perfusion. This not only amplifies the effectiveness of photothermal therapy (PTT) but also facilitates greater intratumoral drug delivery and immune infiltration. The temperature increase also enhances reactive oxygen species (ROS) generation, thereby synergizing with photodynamic therapy (PDT) mechanisms mediated by the DiR molecule. A particularly significant outcome of the ROS cascade is its immunological impact. The increased oxidative stress promotes immunogenic cell death (ICD), leading to the release of damage associated molecular patterns (DAMPs) such as calreticulin and HMGB1. These DAMPs activate the cyclic GMP AMP synthase (cGAS)–stimulator of interferon genes (STING) pathway in antigen presenting cells. As a result, dendritic cells (DCs) mature and prime cytotoxic T lymphocytes (CTLs), mounting a systemic anti-tumor immune response that may suppress both primary tumors and distant metastases.

In summary, the diagram not only visualizes the structural complexity and synthetic methodology of the NanoMn GOx PTX system but also encapsulates its multi-dimensional therapeutic strategy. Through the integration of CDT, PTT, PDT, chemotherapy, imaging, and immune activation within a single platform, this nanoplatform embodies the future of precision oncology offering highly localized, image guided, and immunologically engaged treatment for advanced HCC.

### 3.3 Application of manganese based nanomaterials in medical imaging

Manganese-based nanomaterials have gained significant attention in HCC imaging due to their intrinsic paramagnetic properties, particularly the presence of Mn²^+^ ions, which enhance T1-weighted MRI contrast. This makes them highly effective for T1 weighted MRI contrast enhancement (67). Unlike gadolinium-based agents, Mn based materials offer better biocompatibility and lower risk of nephrogenic systemic fibrosis. Importantly, many manganese containing compounds, such as MnO_2_ or manganese carbonate (MnCO_3_), are designed to be “activatable” undergoing redox reactions or pH triggered dissolution within the tumor microenvironment to release Mn²^+^ ions precisely where imaging contrast is needed (68). This site-specific release improves imaging sensitivity while minimizing systemic exposure.

Comparative analyses of different manganese-based formulations indicate that MnO_2_ nanoparticles are particularly effective in the acidic and reductive tumor microenvironment, where they release Mn²^+^ ions *in situ*, shortening T1 relaxation times and enhancing MRI sensitivity. From a mechanistic standpoint, MnO_2_ nanoparticles play a dual role in both imaging and therapy. In the reductive and acidic conditions of the tumor microenvironment—characterized by elevated glutathione (GSH) and H_2_O_2_ levels MnO_2_ is reduced to free Mn²^+^, which shortens the T1 relaxation time and enhances imaging contrast (69). Concurrently, MnO_2_ acts as a catalyst for the decomposition of H_2_O_2_ into oxygen, alleviating hypoxia—a major barrier to photodynamic therapy (PDT) efficacy. This oxygen generating capability restores ROS production during PDT and boosts treatment response (70). Moreover, the acidic tumor environment triggers degradation of the nanoparticle structure, improving payload release (e.g., drugs or immunostimulants), thus linking the imaging signal with therapeutic action—a concept known as “image guided therapy.

In addition to common diagnostic functions, manganese-based nanomaterials are increasingly being used as an important component of multimodal treatment systems. When combined with photothermal therapy (PTT), chemotherapy, and immunomodulators, the insiturelease of Mn²^+^ ions can not only promote MRI tracking but also directly activate the cGAS STING innate immune pathway, enhancing type I interferon production and dendritic cell maturation. Recent studies have suggested that manganese nanoparticles, such as TPA Mn and ROS sensitive NPMn, trigger cGAS STING signaling, increase secretion of pro inflammatory cytokines (TNF α, IL 6, IL 2), promote cytotoxic T lymphocyte infiltration, and reduce immunosuppressive regulatory T cells. Furthermore, when combined with DNA damaging agents or anti PD 1 therapy, these manganese-based systems synergistically remodel the tumor immune microenvironment and improve immunotherapy efficacy (71) (72). Moreover, manganese nanoparticles functionalized with tumor targeting ligands (e.g., folate, RGD peptides) or surface modifiers (e.g., PEG, lipids) exhibit improved tumor selectivity, circulation halflife, and biosafety. Overall, manganese-based nanomaterials offer a highly integrated solution for the diagnosis and treatment of hepatocellular carcinoma. They can serve not only as contrast agents but also as active drugs to trigger or guide therapeutic responses.

## 4 Construction of big data driven precision medical pathways and clinical prospects

### 4.1 Synergistic role of big data and radiomics in optimizing individualized treatment pathways

The integration of multimodal data analysis and radiomics provides key insights for precision treatment of HCC. Radiomics can systematically extract high dimensional quantitative features from MRI and other medical imaging data, capturing tumor heterogeneity, microstructural patterns, and spatial complexity that are not apparent to clinicians. When combined with clinical biomarkers, genomics, transcriptomics, and patient history, these features provide a robust, data driven framework for individualized clinical decision making (41).

ML and DL algorithms play a central role in this framework by processing multi parametric MRI data, including T1, T2, diffusion weighted imaging (DWI), and apparent diffusion coefficient (ADC) maps, and converting them into predictive models for treatment response, recurrence risk, and survival outcomes (73, 74). Feature selection, model training, and validation strategies are highlighted to ensure reproducibility and generalizability across heterogeneous datasets. Although promising, many models remain in the research stage, constrained by limited prospective validation and inter institutional variability (37) (**Table 1**).

**Table 1:** Radiomics and big data integration in precision therapy for hepatocellular carcinoma (HCC).

Component	Function	Application in HCC precision therapy	Representative Metrics/Results
Radiomic Features	Extract quantitative image traits (e.g., texture, shape, intensity)	Identify tumor heterogeneity, microvascular invasion (MVI), or early recurrence	GLCM based entropy, sphericity, wavelet features correlated with MVI (AUC: 0.81)
Multi parametric MRI Data	Provide anatomical and functional imaging (T1, T2, DWI, ADC)	Enable lesion localization, necrosis evaluation, vascular pattern differentiation	ADC histogram features predict recurrence (AUC: 0.74)
Machine learning (ML) Algorithms	Feature selection, classification, risk scoring	Develop predictive models for treatment response, survival, and staging	LASSO for MVI, Random Forest for TACE response (AUCs 0.78–0.85)
Deep learning (DL) Models	Automated feature extraction, data fusion	Integrate imaging with genomics or pathology	CNN based models predict immunotherapy response with >85% accuracy
Clinical Biomarkers	AFP, DCP, liver enzymes, TNM staging	Enhance model stratification and clinical interpretability	AFP + radiomics improves early HCC detection sensitivity from 0.72 to 0.87
Nanoparticle Characteristics	Size, charge, coating, ligand targeting, drug loading/release	Guide drug carrier design for tumor subtype specificity	Smaller (~50nm) PEGylated MnO_2_ shows higher EPR accumulation in HCC xenografts
Integrated Prognostic Models	Combine radiomics, lab tests, genomics, and clinical history	Predict recurrence, survival, or response to therapy	Combined models yield C index >0.80 for 1 year recurrence prediction

This integrated framework underscores the pivotal role of radiomics and big data in enhancing the granularity and precision of HCC management across diagnostic, prognostic, and therapeutic dimensions. By leveraging quantitative imaging features—many of which are imperceptible to human observers—radiomics offers a non-invasive, reproducible, and high throughput methodology to characterize tumor phenotypes. When aligned with ML algorithms, these features can be transformed into robust predictive tools for assessing microvascular invasion, tumor differentiation, or therapeutic response potential, all of which are critical in informing individualized treatment strategies. Importantly, recent studies have suggested that combining radiomics with conventional clinical biomarkers such as AFP or DCP significantly improves the accuracy of early HCC detection and recurrence prediction. For example, multi parametric models integrating arterial phase texture features with AFP levels have shown superior predictive performance (AUC > 0.85) compared to either modality alone (75). Similarly, radiomics signatures extracted from contrast enhanced MRI and DWI sequences have been correlated with immunotherapy outcomes and TACE responsiveness, suggesting their utility in patient stratification and treatment personalization (76).

Beyond predictive modeling, big data driven approaches facilitate the integration of diverse data layers, including genomics, proteomics, histopathology, and therapeutic history, into unified analytical pipelines. This multidimensional perspective enables the construction of comprehensive patient profiles that account for tumor biology, host response, and environmental variables (77). The insights gained can inform not only the selection of optimal therapeutic regimens (e.g., systemic therapy vs. local ablation) but also the design of drug delivery systems, such as nanoparticle size and surface modification, tailored to specific tumor characteristics. Despite this progress, the clinical translation of radiomics and AI based models remains limited by several challenges. These issues include inconsistent imaging protocols between different hospital institutions, a lack of standardized feature definitions, and insufficient relevant datasets (78). Additionally, many ML models have limited interpretability, which are key barriers to clinical adoption of AI models. To overcome these limitations, future research should prioritize multi center collaboration, standardization of radiomics workflows, and the construction of large annotated datasets incorporating longitudinal follow up results (79). Initiatives such as the Imaging Biomarker Standardization Initiative (IBSI) and the establishment of FAIR (Findable, Accessible, Interoperable, Reproducible) data principles in the field of radiomics are critical steps toward enhancing model reproducibility and clinical trustworthiness (80).

The synergistic integration of radiomics, big data analysis, and clinical information has brought significant progress to personalized HCC treatment. As this field continues to evolve, it is expected to transition from retrospective risk assessment to real time clinical decision support. Additionally, it will find comprehensive applications from treatment selection to nanomedicine design and long-term monitoring. Under appropriate validation and regulatory frameworks, precision oncology based on radiomics has the potential to become a key solution for the next generation of clinical diagnosis and treatment in HCC therapy.

### 4.2 Tumor microenvironment modulation and response prediction modeling

The tumor microenvironment (TME) promotes cell invasion, metastasis, immune evasion, and treatment resistance. The TME is a dynamic, highly heterogeneous system characterized by hypoxia, acidic pH, abnormal vascular structures, and abundant immune suppressive cells, which collectively influence therapeutic outcomes. These unique features significantly impair the efficacy of systemic therapies, particularly immunotherapy and photodynamic therapy (81). Therefore, precise characterization and dynamic monitoring of the TME are critical for precision oncology strategies.

Multi parametric MRI techniques, including diffusion weighted imaging (DWI), dynamic contrast enhanced (DCE) imaging, and apparent diffusion coefficient (ADC) mapping, provide non-invasive, quantitative measures of TME related parameters (82). Studies have shown that decreased ADC values may indicate increased cell density due to active tumor proliferation, while higher K^trans values in DCE MRI correlate with vascular permeability and leakage (83).

Radiomics adds an additional analytical dimension by extracting spatial, textural, and heterogeneity features from imaging data that reflect underlying biological processes. When integrated with circulating biomarkers such as VEGF, HIF 1α, and IL 6, radiomics enables stratification of tumors into immunologically “hot” or “cold” categories, guiding immunotherapy selection, including immune checkpoint inhibitors (84) (85).

ML and DL algorithms, including support vector machines (SVM), random forests, and convolutional neural networks (CNNs), have been applied to develop predictive models of treatment response (86). CNN based radiomics models have achieved >85% accuracy in predicting recurrence following TACE (87), while radiogenomics linking MRI texture to TP53 or CTNNB1 mutation status can predict recurrence with precision up to 0.83. These approaches allow dynamic modeling of tumor evolution and real time adjustment of treatment strategies based on TME heterogeneity (88).

Engineered nanomaterials serve as active modulators of the TME. MnO_2_-based nanoparticles not only function as T1 MRI contrast agents but also react with elevated H_2_O_2_ in the TME to generate oxygen, alleviating tumor hypoxia and enhancing photodynamic therapy efficacy (89). Similarly, glucose oxidase (GOx)-loaded nanoparticles induce tumor starvation via glucose oxidation while generating H_2_O_2_, which synergizes with Mn²^+^ ions to trigger chemodynamic therapy (90). These therapeutic effects can be dynamically monitored via MRI or near-infrared fluorescence imaging, creating a theranostic feedback loop between diagnosis and treatment.

Importantly, modulation of TME conditions—such as oxygenation, ROS levels, and acidity—can sensitize tumors to immune activation. For example, these changes can trigger the cGAS–STING pathway, promoting dendritic cell maturation and enhancing cytotoxic T cell infiltration (91). This provides a rational basis for combination therapies involving nanoparticles, immune checkpoint blockade, and targeted therapies. Together, the convergence of radiomics, AI-driven predictive modeling, and TME-responsive nanotechnology paves the way for adaptive, image-guided, and immuno-integrated strategies for HCC treatment, offering a framework for personalized, precision oncology.

### 4.3 Translational prospects and challenges of nano-imaging therapy integration

The integration of nanotechnology with imaging and therapeutic platforms holds immense translational potential in clinical oncology, particularly in the treatment of HCC. Theranostics, which combines diagnostic imaging with therapy, enables simultaneous, image guided diagnosis and treatment through a single nanotechnology platform, providing a promising avenue for personalized and precision HCC therapy (40).

For example, manganes based nanoparticles not only function as T1 MRI contrast agents but also act as reactive oxygen species (ROS) amplifiers in chemodynamic therapy (CDT). Their ability to respond to the tumor microenvironment (e.g., elevated H_2_O_2_ or acidic pH) enables selective drug release, minimizes off target toxicity, and provides a theoretical basis for MRI guided, data driven treatment planning (84).

However, before nanomaterials can be widely applied in clinical settings, several challenges must be addressed:

1. Toxicity control: Accumulation of inorganic nanomaterials in organs such as the liver or spleen can cause long term adverse effects. Optimizing biodegradability, enhancing renal clearance, and employing bioresponsive or biodegradable nanocarriers are crucial for clinical safety.
2. Targeting accuracy: Although ligand modification (e.g., hyaluronic acid or antibodies) improves tumor selectivity, heterogeneity of receptor expression often leads to low delivery efficiency (41). Emerging strategies include adaptive, dual targeting, or stimuli responsive nanoparticles that leverage imaging and radiomics data to enhance site specific delivery.
3. Standardization of imaging and analysis: Significant differences exist among institutions in MRI acquisition protocols, radiomics feature extraction, and image quality (92). This variability limits reproducibility and the reliability of imaging guided therapeutic decisions. Establishing standardized imaging protocols, open source radiomics pipelines, and data sharing frameworks is essential for clinical validation and broader adoption of nanomedicine platforms.

Currently, integrated nano imaging and therapeutic systems suggeste substantial potential for advancing HCC management. By combining radiomics, AI driven predictive modeling, and TME responsive nanomaterials, clinicians can implement adaptive, image guided treatment regimens that account for tumor heterogeneity, predict therapeutic response, and optimize combination strategies.

Translating these approaches from research to clinical practice requires addressing toxicology, targeting efficiency, and standardization challenges. Successful integration of big data analytics, standardized imaging, and nanomedicine will enable safe, precise, and personalized HCC therapy, ultimately expanding treatment options and improving patient outcomes.

## 5 Conclusion and the future directions

### 5.1 Conclusion

The comprehensive application of nanomaterials, radiomics, and big data technology in the precise diagnosis and treatment of hepatocellular carcinoma (HCC) has emerged as a prominent research frontier in recent years. By critically evaluating the latest advancements in manganese-based nanoparticles for MRI enhancement and multimodal synergistic therapy—including chemotherapy, chemodynamic therapy (CDT), photothermal therapy (PTT), and photodynamic therapy (PDT)—this review highlights the dual diagnostic and therapeutic roles of nanomedicine. The application of these multifunctional nanoplatforms enables real-time tumor localization, dynamic treatment response monitoring, and image-guided intervention, collectively driving enhanced precision in therapeutic decision-making.

Furthermore, the integration of radiomics provides critical quantitative insights into tumor heterogeneity, microenvironmental changes, and functional dynamics that are otherwise undetectable by conventional imaging. When combined with machine learning algorithms, these radiomic features can be harnessed for predictive modeling of treatment response and individualized therapy planning. The fusion of imaging biomarkers with clinical and molecular data facilitates the development of adaptive, closed-loop treatment workflows, supporting personalized and evidence-based clinical decision-making.

The tumor microenvironment (TME) remains a critical determinant of treatment efficacy, influencing both imaging outcomes and therapeutic responses. Predictive modeling incorporating molecular markers, imaging data, and nanomaterial-induced microenvironmental modulation can enhance treatment adaptability and precision. However, significant challenges remain for clinical translation, including systemic toxicity, off-target effects, limited targeting accuracy, and the lack of standardized protocols for imaging and radiomic analysis. These limitations underscore the need for interdisciplinary research, rigorous preclinical validation, and carefully designed clinical trials.

In summary, this review provides a comprehensive synthesis of the immense potential of nanomaterial-enabled radiomics and big data-driven models in HCC diagnosis and therapy. Looking forward, the convergence of advanced imaging technologies, intelligent data modeling, and multifunctional nanotherapeutics promises to establish a new era of personalized, image-guided, and data-driven medicine for HCC, ultimately improving patient outcomes and enabling precision oncology at scale.

### 5.2 Future directions

Despite significant advances in the application of nanomaterials for HCC imaging and therapy, several research gaps and challenges remain that warrant further investigation. First, although manganese-based and other functionalized nanoparticles have suggested promising preclinical imaging performance and therapeutic potential, their clinical translation is limited by issues such as systemic toxicity, long-term metabolism, and heterogeneous tumor uptake. Future studies should focus on optimizing nanoparticle design, including size, surface chemistry, and targeting ligands, to maximize tumor specificity and biosafety.

Second, integration of nanomaterials with radiomics and big data-driven predictive modeling remains in its early stages. While preliminary studies suggest that combining quantitative imaging features with machine learning can enhance individualized treatment planning, standardized protocols for data acquisition, feature extraction, and validation across multiple centers are urgently needed to ensure reproducibility and clinical applicability.

Third, the tumor microenvironment and immune response play critical roles in both imaging performance and therapeutic efficacy. Future research should explore multimodal nanomaterials capable of modulating hypoxia, ROS levels, or immune pathways (e.g., cGAS-STING activation) to enhance both diagnostic accuracy and treatment response. Mechanistic studies linking nanoparticle behavior with microenvironmental factors will be crucial for rational design of next-generation theranostic platforms.

Finally, translational studies bridging preclinical models and human patients are essential. Large-scale, well-controlled clinical trials, along with regulatory standardization and long-term safety assessments, will be necessary to realize the full potential of nanotechnology-enabled precision medicine in HCC.

In summary, future directions should emphasize rational nanoparticle design, integration with radiomics and AI, tumor microenvironment modulation, and rigorous translational evaluation, thereby paving the way for more precise, safe, and effective HCC diagnosis and therapy.
